# DetSpace: a web server for engineering detectable pathways for bio-based chemical production

**DOI:** 10.1093/nar/gkae287

**Published:** 2024-04-18

**Authors:** Hèctor Martín Lázaro, Ricardo Marín Bautista, Pablo Carbonell

**Affiliations:** Institute of Industrial Control Systems and Computing (AI2), Universitat Politècnica de València (UPV), Camí de Vera s/n, 46022 València, Spain; Institute of Industrial Control Systems and Computing (AI2), Universitat Politècnica de València (UPV), Camí de Vera s/n, 46022 València, Spain; Institute of Industrial Control Systems and Computing (AI2), Universitat Politècnica de València (UPV), Camí de Vera s/n, 46022 València, Spain; Institute for Integrative Systems Biology I2SysBio, Universitat de València-CSIC, Escardino Street 9, Paterna, 46980 València, Spain

## Abstract

Tackling climate change challenges requires replacing current chemical industrial processes through the rational and sustainable use of biodiversity resources. To that end, production routes to key bio-based chemicals for the bioeconomy have been identified. However, their production still remains inefficient in terms of titers, rates, and yields; because of the hurdles found when scaling up. In order to make production more efficient, strategies like automated screening and dynamic pathway regulation through biosensors have been applied as part of strain optimization. However, to date, no systematic way exists to design a genetic circuit that is responsive to concentrations of a given target compound. Here, the DetSpace web server provides a set of integrated tools that allows a user to select and design a biological circuit that performs the sensing of a molecule of interest by its enzymatic conversion to a detectable molecule through a transcription factor. In that way, the DetSpace web server allows synthetic biologists to easily design biosensing routes for the dynamic regulation of metabolic pathways in applications ranging from genetic circuits design, screening, production, and bioremediation of bio-based chemicals, to diagnostics and drug delivery.

## Graphical Abstract

**Graphical Abstract:**
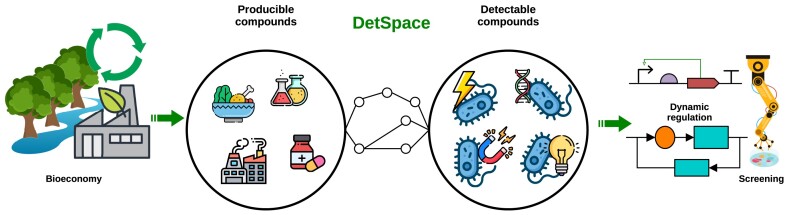

## Introduction

Biomanufacturing initiatives are currently thriving worldwide to address the societal challenges and threats posed by climate change (1). Policymakers and global stakeholders have established bioeconomy goals to achieve carbon neutrality in the next decade (2). A well-established objective towards that end is to replace current production processes, for a substantial amount of key compounds in the consumer value chain (which are primarily carried out by the chemical industry based on fossil resources), by greener biomanufacturing processes (3). In the last years, metabolic engineering efforts combined with prospective studies based on retrobiosynthetic tools (4) have identified biosynthetic routes for production of chemicals of industrial interest (5). However, most of the known routes have only been validated at small volumes. Therefore, in order to achieve industrial levels, it would be necessary to ensure that their bioproduction processes are still efficient and reproducible in terms of titer, yield, and rate (6,7).

Achieving desirable strains with optimal production, at fermentation levels, requires both an efficient way for high-throughput screening and a strategy for pathway robustness against context-dependency uncertainty, notably by engineering dynamic regulation in the production pathway (8). Transcription factor-based biosensors, which are genetic circuits that can detect the concentration of the target compound and elicit a response, provide a promising approach for achieving such goal (9,10). However, the number of known molecules that can act as effectors for allosteric transcription factors is limited (11). To alleviate this limitation, a strategy has been proposed based on the use of extended biosensors. The strategy consists of a pathway where through enzymatic conversions the target compound is transformed into a detectable compound (12,13); defining in that way an extended metabolic detectable space for the target compounds. The extent of such space is yet to be charted, and its determination is nowadays crucial and instrumental for the bioeconomy. This is due to the fact that this specific knowledge will substantially improve our understanding of the biomanufacturing potential capabilities for bio-based chemicals.

Here, we present DetSpace: a web server that allows a user to navigate through a comprehensive map of bio-based products of industrial interest, and to select (in a desired microbial chassis) their associated genetic circuits consisting of detectable pathways; thus leading to known effectors, as well as their associated transcription factors. Integrating the DetSpace extended detectable pathway design as part of the Design-Build-Test-Learn pipeline of biomanufacturing and biofoundries contributes to more robust and efficient bio-based production; bringing closer the fulfillment of the goals fostered by the future bioeconomy.

## Materials and methods

### Datasets

#### Chassis selection

A selection of genome-scale models from conventional and alternative chassis at lab and industrial scale (14) were compiled and their metabolomes were obtained from the BiGG database (15) or the BioCyc collection (16).

#### Producible compounds

The set of target compounds of industrial interest was obtained from a curated map of bio-based chemical compounds (5).

#### Detectable compounds

The list of detectable molecules and their associated transcription factors was obtained from Sensbio, a comprehensive database of biosensors based on allosteric transcription factors (11).

#### Bioretrosynthesis

The set of reaction rules RetroRules (17) was used to connect detectable to producible compounds using bioretrosynthesis. Such set can be tuned in terms of specificity by selecting the diameter, which is a parameter that determines the description of the reaction based on atom vicinity around the reaction center.

### Calculation of detectable routes

Detectable routes connecting bio-based compounds were determined in the extended metabolic space through a four-step bioretrosynthesis procedure as detailed in the following.

#### Metabolic expansion

The RetroRules database allows the application of a generative algorithm for expanding the metabolic space by an iterative backward prediction of compounds that can be used as reactants, starting from the detectable compounds.

#### Monte Carlo tree search

Since the metabolic expansion is highly combinatorial, an artificial intelligence approach based on Monte Carlo tree search was used to limit the search (18). The Monte Carlo was launched for each pair of detectable and producible compounds. At each step, the new nodes of the tree are scored using Tanimoto similarity with the target molecule in order to guide the search. See Supplementary Note 1 for a detailed explanation of the algorithm.

#### Pathway enumeration

Once a producible compounds is reached, the set of detectable pathways is determined by combining all the shortest pathways for each branched node within the scope of biochemical transformations linking to the detectable compound. See Supplementary Note 2 for a detailed explanation of the algorithm.

#### Pathway ranking

Reaction rules in the RetroRules database have a penalty score, which was used to rank the pathways by selecting the higher individual score as the global score. Moreover the estimated steady-state flux of the production pathway precursors was included in the ranking calculation.

### Calculation of producible routes

Starting from a comprehensive metabolic map of bio-based compounds of industrial interest (19), major natural and engineered production pathways were determined, linking the compounds to the central metabolism of the chassis. The map contains a total of 532 producible compounds and 438 biological reactions (enzyme-catalyzed reactions), which were further processed for detailed annotation and cross-link database references.

### Genetic design

Each chemical reaction included in the pathways generated by DetSpace contains a link to query the Selenzyme web server (20), a free online enzyme selection tool for metabolic pathway design. This search provides the user with additional information about the enzymes that catalyze specific reactions in different chassis organisms, in order to choose the best candidates for a given pathway step; thus allowing a more accurate and rational enzyme selection. Furthermore, the resulting pathway can be exported into JSON Cytoscape.js (21) format and as SBML, allowing further processing for the generation of the genetic circuit associated with the pathway (see Supplementary Figure S1) using genetic design tools, such as the Galaxy SynBioCAD workflow (22).

### Implementation

#### DetSpace web development

The DetSpace web server runs on the Django platform in Python 3.11, using the JQuery framework for the front end interface and the Cytoscape.js library (21) for the visualization of the metabolic networks and pathways. The code is freely available at https://github.com/dbtl-synbio/detspace.

#### API features

DetSpace provides an API based on the REST framework with the following functionalities: retrieval of the list of chassis organisms; producible and detectable compounds and its corresponding paired combinations; information regarding the transcription factors associated with each detectable compound; and the list of detectable pathways in both SBML and JSON format.

## Description of the web server

**Figure 1.**
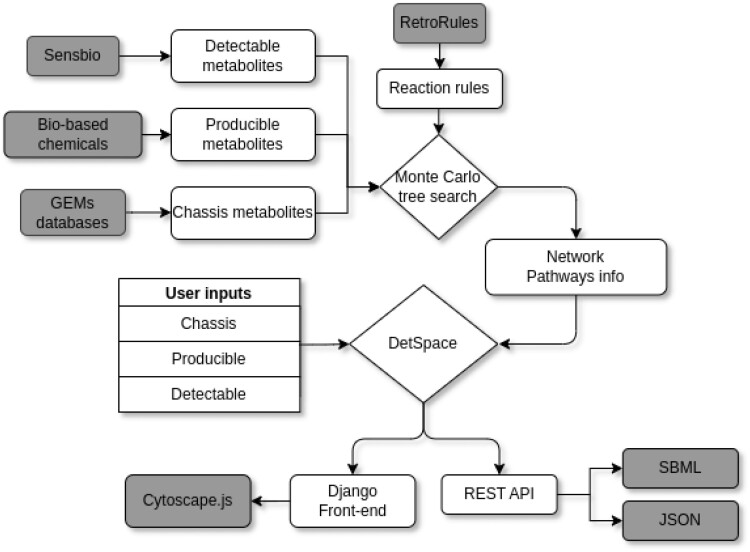
Workflow of the DetSpace functionality and data sources. The set of detectable and producible metabolites go through a Monte Carlo tree search algorithm in order to generate all pathways. This information is combined on the webserver with the user-selected chassis, producible and detectable compounds.

Figure 1 shows the DetSpace workflow and data sources. The entry point of the web server is a target compound selected from a list of bio-based chemicals of major interest for metabolic engineering (19) and the desired microbial host as industrial chassis. The server calculates the different alternative steps needed to convert the target chemical into one of the effector molecules reported in the literature that can be detected through a transcription factor, as well as the required precursors in the pathway. The design process consists of the following steps:

### Chassis selection

The user can select the chassis organism where the pathway will be engineered from two lists: one containing a taxonomic selection of main industrial and prototyping chassis used by the synthetic biology and metabolic engineering community classified; and the other one grouped by application. The selected chassis will determine the number of endogenous and heterologous metabolites present in the pathway. Typically, a DetSpace pathway should contain a high ratio of heterologous metabolites in order to avoid cross-talk and flux diversion and, therefore, this consideration might determine in some cases the selection of the appropriate chassis.

### Target selection

The user selects the target compound of interest from a list that contains a comprehensive metabolic map for production of bio-based chemicals. DetSpace provides a curated list of bio-based chemicals that are essential building blocks and compounds for the bioeconomy, based on the metabolic map collected by Lee *et al.* (5). Only those compounds associated with detectable pathways are available for selection in the list.

### Effector selection

The next step is the selection of the effector compound of a transcription factor that can be connected to the target product. A curated list of detectable molecules that can act as allosteric effectors for transcription factors is provided based on the molecule-transcription factor pairs database collected by Carbonell and colleagues in 2023 (11). For each selected target compound, only those effectors that can be associated through a DetSpace pathway to the compound are shown in the list.

### Retrosynthetic analysis

Retrosynthetic routes for production of the precursors in the chassis are graphically displayed, as well as required supplements to add to the growth media. The routes are calculated based on a Monte Carlo tree search algorithm that prioritizes pathways involving less complex biochemical transformations from the RetroRules database of reaction rules. Each given solution pair of target product and effectors can lead to multiple alternative pathways. A prioritization list is provided based on the reaction rules scores. The pathways are displayed graphically on an interactive map where the user can select individual pathways and explore in more detail the intermediates in the pathway, as well as the information about each enzymatic transformation.

### Production routes design

The main backbone upstream routes for bio-based production of the target compound can be accessed. Main production routes of bio-based compounds are provided based on their metabolic map, which can be further analyzed through the use of bioretrosynthesis tools such as RetroPath 2.0 (23).

### Detectable routes design

DetSpace provides the list of enzyme sequences, selected through the Selenzyme algorithm (20) and the combinatorial library of the genetic circuits for building the detectable pathway. The selected genes and pathways can be exported into SBML format so that they can be transferred to the Galaxy SynBioCAD genetic design workflow (22), in order to build and assembly a combinatorial library for further engineering and optimization of the DetSpace detectable pathway for bio-based production.

## Results

### Overall results

Overall results are shown on Table 1. In total, 10 231 producible-detectable pairs were identified that can be connected through 914 192 pathways. The metabolic scope contains 2227 intermediate compounds, 1042 precursors (molecules that are in the chassis or at least a production pathway exists to produce it from the chassis according to the literature (5) or the retrosynthetic analysis (23), 1959 supplement compounds that need to be added to the media in order to make viable some of the pathways. These results were calculated for the Escherichia coli chassis. Table 2 shows chassis-independent specific numbers for each type of compound. For the producible set, there are 198 products with available DetSpace pathway, with and average number of pathway per product of 4617 and an average number of pairs close to 52. For the detectable set, there are 303 effectors with available DetSpace pathway, with and average number of pathway per effector of 3017 and an average number of pairs close to 34.

**Table 1.** Overall results (for *Escherichia coli*)

	Pairs	Pathways	Intermediates	Precursors	Supplements
Total	10 231	914 192	2227	1042	1959

**Table 2.** Results by compound

	Number of	Average no.	Average no.
	compounds	of pathways	of pairs
Producible	198	4617	51.67
Detectable	303	3017	33.76

### Case study 1

**Figure 2.**
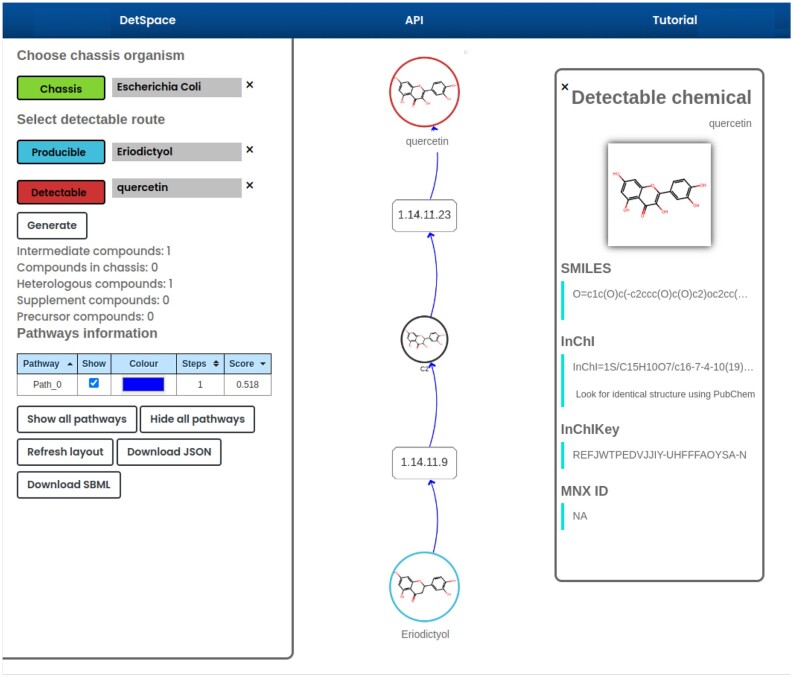
Detectable pathway for the (2*S*)-eriodyctiol to quercetin.

(2*S*)-Eriodyctiol is a flavonoid belonging to the flavanones subclass that is widespread in citrus fruits, vegetables, and medicinally important plants, and with important antioxidant and anticancer properties (24). Eriodyctiol is an hydroxylated product of (2*S*)-naringenin and therefore its production is of interest in the production of high-value molecules from agricultural waste. Detecting eriodyctiol through a biosensor circuit will therefore open also the possibility for indirect sensing of naringenin, with potential applications for screening and dynamic regulation (12), including directed evolution for efficient production (25). According to DetSpace, eriodictyol can be downstream transformed into two molecules with associated allosteric transcription factor, i.e., into quercetin, which can be detected by the transcription factor LmrA from *Bacillus subtilis* (26) by a two-step transformation involving taxifolin (see Figure 2), and into catechin by a three-step transformation (see Supplementary Figure S2), which can be detected by the transcription factor QdoR from *Bacillus subtilis* (26), belonging both to the repressor TetR family.

### Case study 2

Campesterol is a plant sterol with cholesterol lowering and anticarcinogenic effects (27). Interestingly, multiple pathways were found by DetSpace converting campesterol into acyl-CoA thioesters, like feruloyl-CoA (4 pathways), caffeoyl-CoA (12 pathways), shown in Supplementary Figure S3, oleoyl-CoA and palmitoyl-CoA (24 pathways), which are fatty esters involved in the biosynthesis of flavonoids and various alkaloids. Several species have developed allosteric transcription factors related to those compounds to regulate fatty acid metabolism, such as FadR (28) and FerC (29), in the case of feruloyl-CoA. Detection pathways linking campesterol to those effectors were found containing between five and seven steps.

## Conclusions and future perspectives

Determining the extended detectable biochemical space of key bioeconomy bio-based compounds through DetSpace has a major utility, as it allows to quickly visualize and explore biosensing pathways for screening and dynamic regulation that are expected to become instrumental in order to scale up production for a substantial amount of bio-sourced compounds into industrial levels. In the near future we plan to incorporate to the DetSpace server a predictive machine learning model of biochemical properties of compounds with industrial interest (pharmacological, aromatic, toxic potential...), and to automate the design of the detectable pathways under desired specifications for dynamic and operating ranges.

## Supplementary Material

gkae287_Supplemental_File

## Data Availability

DetSpace is an open-source application, available at GitHub (https://github.com/dbtl-synbio/detspace) and FigShare at https://doi.org/10.6084/m9.figshare.25103978. The DetSpace website is free and open to all users, there is no login requirement, available at https://detspace.carbonelllab.org/.
