# Supplementary material for: DetSpace: a web server for engineering detectable pathways for bio-based chemical production
Source: Nucleic Acids Res. 2024 Apr 18;52(W1):W476–80. doi: 10.1093/nar/gkae287 (PMC11223873; doi:10.1093/nar/gkae287)
Supplement: gkae287_Supplemental_File [file gkae287_supplemental_file.pdf]

DetSpace Supplementary Information

Authors: Hèctor Martín Lázaro[1], Ricardo Marín Bautista[1], Pablo Carbonell[1,2]

Title: DetSpace: A web server for engineering detectable pathways for bio-based chemical production

Affiliation: [1]Institute of Industrial Control Systems and Computing (AI2), Universitat Politècnica de València (UPV), 46022 València, Spain and [2]Institute for Integrative Systems Biology I2SysBio, Universitat de València-CSIC, Escardino Street 9, Paterna, 46980 València, Spain

Contents

- Supplementary Note 1. Monte Carlo tree search algorithm 1
- Supplementary Note 2. Pathway enumeration algorithm 2
- Supplementary Figure 1. Genetic design example. 3
- Supplementary Figure 2. Catechin example. 4
- Supplementary Figure 3. Campesterol example. 5

1. Supplementary Note 1. Monte Carlo tree search algorithm:

As mentioned in the article, a Monte Carlo tree search algorithm has been used to obtain the detectable routes. In this section, we are going to detail how it works.

Input: First, we start from the producible and detectable pair, plus the reaction rules of RetroRules. These rules allow us to generate new metabolites until we obtain the complete route. The Python module *RDKit*, which specializes in cheminformatics, has been used for this purpose.

Short description: The process to generate the new metabolites consists of launching our detectable against all the rules and writing down the new compounds obtained. Here is where the Monte Carlo algorithm comes into play, as this is an exponential expansion, so it is necessary to limit the search. Each new generation of metabolites adds more paths to the search tree and the aim of the algorithm is to find the most promising paths. It consists of four steps: selection, expansion, simulation, and backpropagation.

Steps:

1. **Selection:** Starting from the root node, we navigate through the tree until we reach the leaf nodes that have not yet been simulated. The nodes with the highest assigned score are chosen for this traversal, so that we always take the most promising path.
2. **Expansion:** Once the node is selected, new compounds are generated using the rules. It is possible that these new metabolites are already in the tree, when this happens, they are simply omitted. The rest are added as child nodes of our selected compound. In case the selected node has a score of 1, we have already reached our target compound, so the algorithm would end here.
3. **Simulation:** For all new nodes a score is calculated. This score consists of the Tanimoto similarity with the target metabolite.
4. **Backpropagation:** Using the score of the new nodes, the information of the nodes on the path between the root and these new nodes is updated. To prevent the algorithm from delving too deeply into some branches and ignoring other promising ones, a correction factor has been added to the score. Nodes that have not been chosen for a long period of iterations gradually increase their score, so that at some point the algorithm will change branches and explore the others.
5. **Output:** When the search has reached our target compound the algorithm returns the resulting tree and a file containing info from all the reactions taking part on it.

The computations were performed on the HPC cluster Garnatxa at Institute for Integrative Systems Biology (I2SysBio), I2SysBio is a mixed research center formed by University of Valencia (UV) and Spanish National Research Council (CSIC), and at the HPC cluster Rigel of the Universitat Politècnica de València.

2. Supplementary Note 2. Pathway enumeration algorithm:

Input: in order to enumerate the pathways, the algorithm takes the output from the Monte Carlo tree search.

Short description: The pathway enumeration algorithm is mainly based on functions of the Python module NetworkX, which is a package focused on working with networks. The algorithm can be divided into three stages, the creation of the graph, the search for pathways and the addition of branched pathways.

Steps:

1. **Creation of the graph:** with the data obtained through Monte Carlo we generate a bipartite graph where the nodes can be the compounds or the reactions that relate them. This graph is inverted with respect to the Monte Carlo tree. In the metabolic expansion we start from the detectable and by bioretrosynthesis we arrive at the producible. However, our goal is the reverse route, from producible to detectable, so this first step needs to invert the tree.
2. **Search for pathways:** the NetworkX functions are used to obtain a list of all possible simple routes, which means routes that are not cyclic.
3. **Branched pathways:** we have to add what we call branched pathways. The reaction rules of RetroRules are annotated in such a way that there is only one substrate, but in reality these rules may need several substrates. The algorithm navigates the network in search of these reactions with several substrates. Once located, the missing substrates are added to the network and the corresponding pathways.

Output: the algorithm returns a list with the complete pathways enumeration.

3. Supplementary Figure 1. Genetic design example:

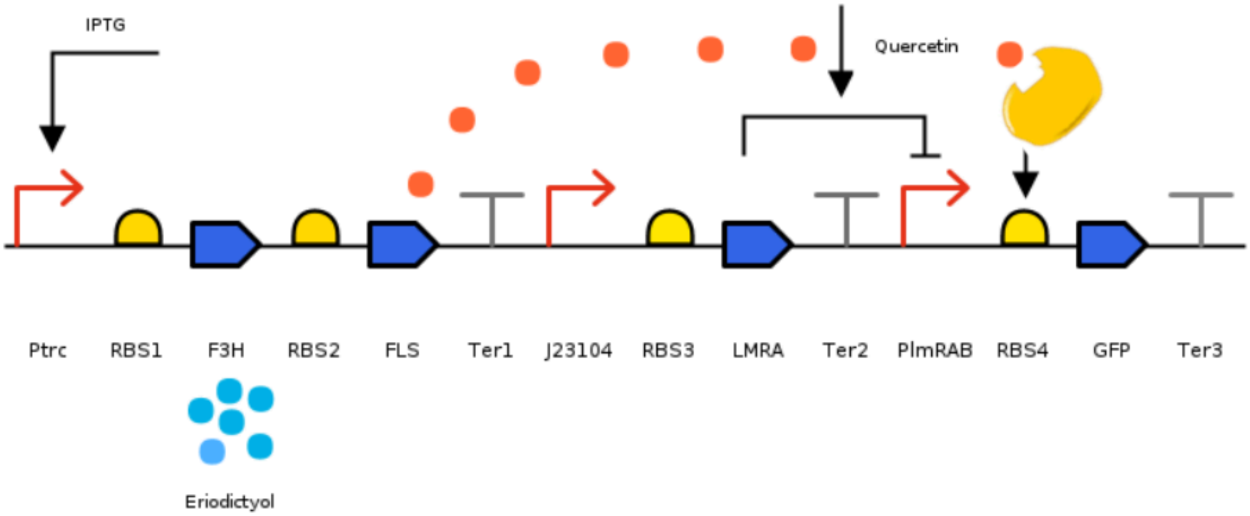

Figure S1: According to DetSpace, eriodictyol can be downstream transformed into quercetin with associated allosteric transcription factor by a two-step transformation involving taxifolin; which can be detected by the transcription factor LmrA from Bacillus subtilis. This process can be detected by the transcription factor QdoR from Bacillus subtilis, belonging both to the repressor TetR family.

4. Supplementary Figure 2. Catechin example:

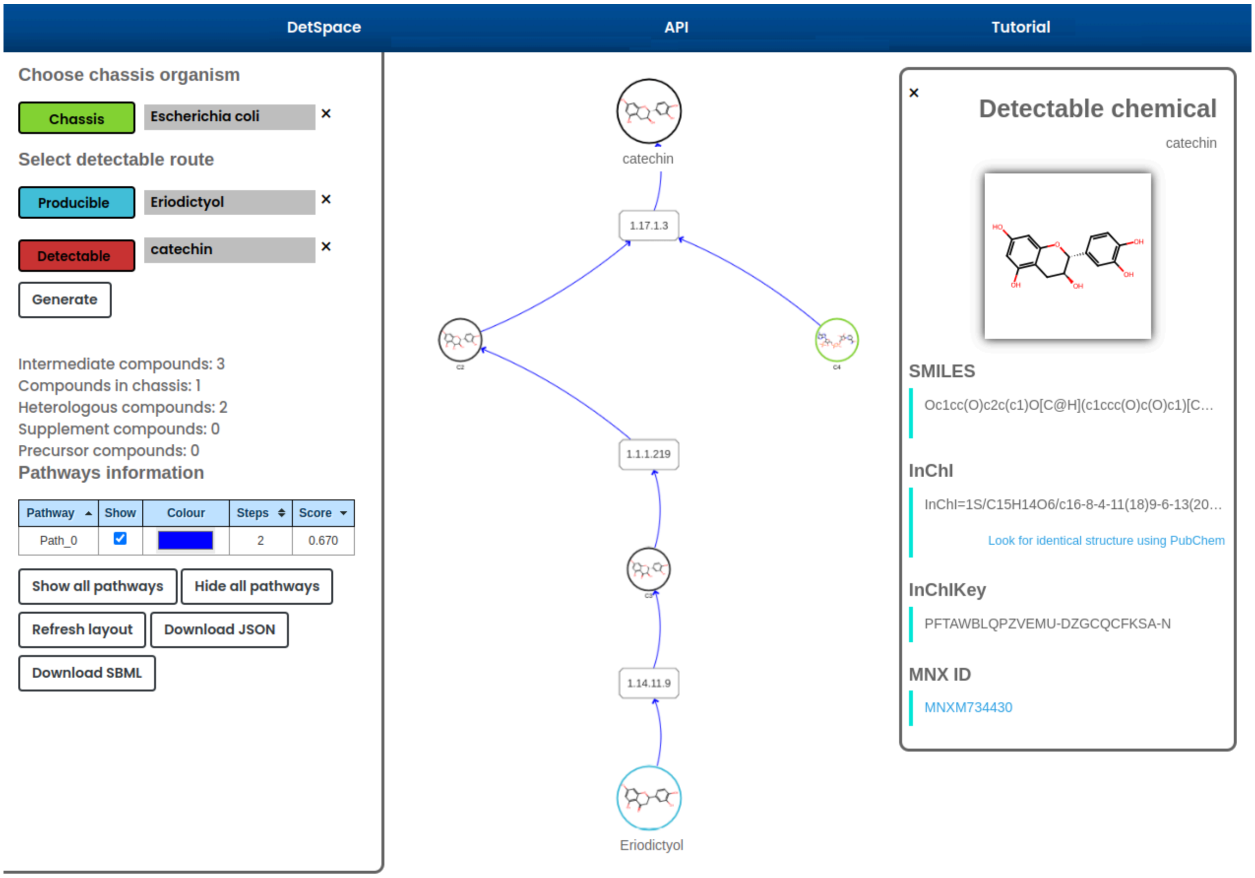

Figure S2: According to DetSpace, eriodictyol can be downstream transformed into catechin, which e.g. can be detected by the transcription factor LmrA from *Bacillus subtilis*, by a three-step transformation involving taxifolin and leucocyanidin.

5. Supplementary Figure 3. Campesterol example:

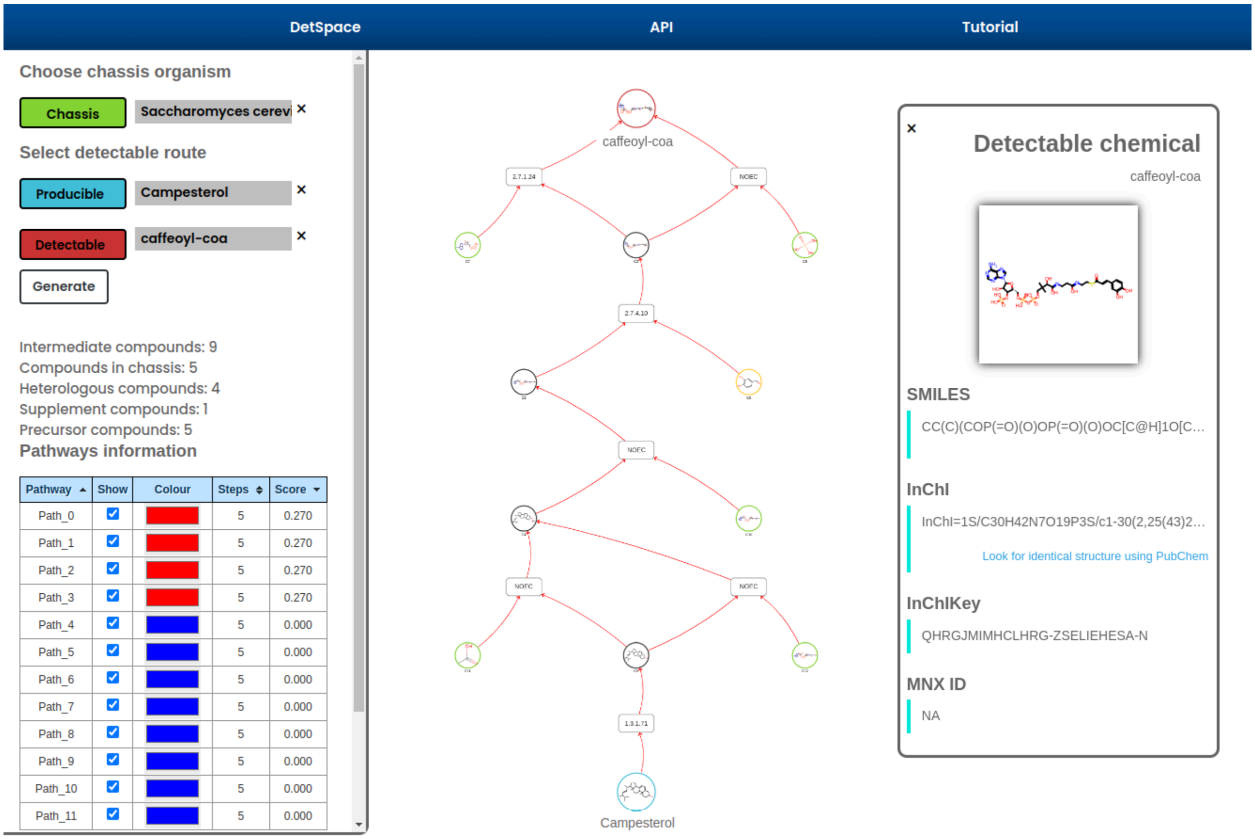

Figure S3: According to DetSpace, campesterol can be downstream transformed into caffeoyl-CoA by a five-step transformation involving eleven different pathways with slightly different combinations of enzymes and compounds.
